# Correlation between peripheral blood inflammatory indicators and pathologic complete response to neoadjuvant chemotherapy in locally advanced breast cancer patients

**DOI:** 10.1097/MD.0000000000020346

**Published:** 2020-05-29

**Authors:** Tulay Eren, Cengiz Karacin, Gokhan Ucar, Yakup Ergun, Ozan Yazici, Goksen İnanc İmamoglu, Nuriye Ozdemir

**Affiliations:** a Dişkapi Yildirim Beyazit Research and Education Hospital; b Ankara City Hospital; c Gazi University, Department of Medical Oncology, Ankara, Turkey.

**Keywords:** breast cancer, lymphocyte-to-monocyte ratio, neoadjuvant chemotherapy, neutrophil-to-lymphocyte ratio, pathologic complete response, platelet-to-lymphocyte ratio

## Abstract

The immune system plays a fundamental role in the response to neoadjuvant chemotherapy (NAC) of locally advanced breast cancer (LABC) patients. Patients with pathological complete response (pCR) after NAC have a higher survival rate. Neutrophil-to-lymphocyte ratio (NLR), platelet-to-lymphocyte ratio (PLR), and lymphocyte-to-monocyte ratio (LMR) are peripheral blood indicators of inflammatory response. This investigates the correlation between NLR, PLR, LMR, and other clinicopathological features of breast cancer patients before receiving NAC and pCR.

Data of LABC patients who underwent NAC between 2009 and 2018 were retrospectively reviewed. Each patient's peripheral complete blood count was recorded before starting NAC. The cut-off values for neutrophils, lymphocytes, monocytes, and platelets in the peripheral blood and NLR, PLR, and LMR were determined by receiver operating characteristic curve analyses.

The records of 131 patients were analyzed and divided into two groups, pCR (+ve) and pCR (−ve), and their clinicopathological features and laboratory findings were compared. pCR was achieved in 23.6% of patients. The cut-off values of neutrophils, lymphocytes, monocytes, and platelets at the time of diagnosis and NLR, PLR, and LMR were, respectively, 4150 μL, 2000 μL, 635 μL, 271 × 10^3^ μL, 1.95, 119, and 3.35. The pCR rate was higher in patients with low neutrophil count, low NLR, and high lymphocyte count (*P* = .002, <.001, and .040, respectively).

As per the findings of multivariate logistic regression analysis, the independent predictive factors of pCR were clinical tumor size T1 and T2, grade 3, ER negativity, and low NLR (*P* = .015, .001, .020, .022, and .001, respectively).

While NLR was found to be an independent predictive factor of pCR in LABC patients receiving NAC, a similar result was not observed for PLR and LMR. NLR can be a useful biomarker for predicting the response of patients receiving NAC.

## 1 Introduction

Neoadjuvant chemotherapy (NAC) is the standard treatment modality for locally advanced breast cancer (LABC).^[1]^ There is no distinctive difference in disease-free survival (DFS) and overall survival (OS) between NAC and adjuvant chemotherapy.^[1]^ However, the OS is found to be higher for patients in whom pathologic complete response (pCR) is achieved after NAC than for patients in whom pCR is not achieved.^[2,3]^ Therefore, numerous studies have been looking into the clinicopathological features that can be used to predict pCR in patients receiving NAC.^[4]^ It is known that pCR is better in patients who have human epidermal growth factor receptor 2 (HER-2) positive or triple negative breast cancer compared to patients with HER-2 negative or hormone receptor positive breast cancer.^[4]^ Clinical tumor size (cT) and tumor grade are other clinicopathological features that are used to predict pCR.^[4,5]^ Apart from clinicopathological features, some studies showed that genetic and molecular factors can be used to predict NAC response.^[6,7]^ The cost, complexity, and limited access to genetic and molecular tests in many countries complicate their usage in daily practice. Thus, simpler and easily accessible pathologic or laboratory markers are needed to predict NAC response.

Researchers have demonstrated that inflammatory markers in peripheral blood and immune-related indicators predict survival and chemotherapy response in different types of tumors.^[8–10]^ Survival is negatively affected by a high number of neutrophils and platelets and low number of lymphocytes prior to treatment in cancer patients.^[11]^ Some studies showed that neutrophil–lymphocyte ratio (NLR), platelet–lymphocyte ratio (PLR), and lymphocyte–monocyte ratio (LMR), which are easily calculated factors of systemic inflammatory response, are prognostic factors in breast cancer.^[12–15]^ Following the studies which showed that NLR, PLR, and LMR are prognostic factors in breast cancer, their correlation with pCR in locally advanced cancer patients receiving NAC has been investigated. Studies have demonstrated that the pCR rate is higher for patients with low NLR than for those with high NLR.^[16,17]^ Contrary to these results, some studies imply that there is no correlation between NLR and pCR.^[18,19]^ A limited number of studies has looked into the relation between PLR and neoadjuvant response. Asano et al found that low PLR is an independent predictive factor of pCR.^[20]^ In a study investigating the correlation between LMR and pCR, LMR was found to be an independent predictive factor of pCR.^[5]^

The aim of this study was to determine the clinicopathological features and systemic inflammatory indicators predictive of pCR after NAC in LABC patients.

## 2 Materials and methods

### 2.1 Patients

Data of patients who received NAC after being diagnosed with LABC in the Medical Oncology Clinic of Ankara Numune Training and Research Hospital between 2009 and 2018 were retrospectively reviewed. The inclusion criteria were as follows: female gender, clinical status of II to III according to the 7th Edition tumor-node-metastasis (TNM) classification of the American Joint Committee on Cancer Staging (AJCC),^[21]^ complete blood count performed prior to NAC, completion of NAC, surgical treatment after NAC, and availability of postoperative pathology reports. As part of the NAC regimen, the patients were administered anthracycline-based regimens (4 cycles of doxorubicin 60 mg/m^2^ and cyclophosphamide 600 mg/m^2^, or cyclophosphamide 600 mg/m^2^ and epirubicin 50 mg/m^2^, every 3 weeks) and taxane-based regimens (paclitaxel 80 mg/m^2^ for 12 weeks or 4 cycles of docetaxel 75 mg/m^2^, every 3 weeks). Trastuzumab along with taxane was administered to HER-2-positive patients. Following NAC, the patients underwent mastectomy or axillary lymph node dissection with breast-conserving surgery or sentinel lymph node biopsy. The exclusion criteria included male gender, antibiotic use within the last week before NAC, corticosteroid use, presence of rheumatological diseases, existing chronic liver or chronic renal diseases, breast cancer diagnosis during pregnancy, failure to complete NAC, and absence of surgical treatment.

### 2.2 Subtypes of breast cancer

Histological type, size, and grade of the tumor and level of lymph node involvement were examined for all the patients included in the study. The specimens of needle biopsy performed prior to NAC and on the tissues after surgery were subjected to histopathological and immunohistochemical (IHC) examinations.

Estrogen receptor (ER), progesterone receptor (PR), and HER-2 status were determined by the IHC method; the specimens of patients with a staining level of <1% in the tumor cells were considered as having negative ER and PR status; further, HER-2 status was regarded positive if it was ≥3+ and negative if it was ≤1+. Then, HER-2 status was confirmed by fluorescence in situ hybridization (FISH) or silver in situ hybridization (SISH) studies for patients with 2+ HER-2 status on IHC testing. Tumor grade was defined as grade 1, grade 2, or grade 3 according to the Scarff-Bloom-Richardson classification. Breast cancer was classified into four subtypes: luminal A, luminal B/HER-2 positive, luminal B/HER-2 negative, HER-2 positive, and triple-negative.

### 2.3 Tumor response

In the postoperative pathological evaluation, the absence of invasive tumors in the breast tissue or lymph node (independent of the presence of an in situ component) was defined as pCR (ypT0/ypN0).

### 2.4 Blood samples and data collection

Peripheral complete blood count was performed before administering NAC. Neutrophil, lymphocyte, platelet, and monocyte counts, and all laboratory indexes were evaluated prior to starting NAC. NLR was calculated as the rate of absolute neutrophil count to absolute lymphocyte count, PLR, as the rate of absolute platelet count to absolute lymphocyte count, and LMR, as the rate of absolute lymphocyte count to absolute monocyte count.

Body mass index (BMI) was calculated as body mass (kg) divided by height (m) squared. A BMI of 18.5 to 24.9 was considered normal, 25 to 29.9, overweight, 30 to 34.9, obese class 1, 35 to 44.9, obese class 2, and 45 and higher, obese class 3.

Approval was obtained from the ethics committee of Dişkapi Yildirim Beyazit Training and Research Hospital.

### 2.5 Statistical analyses

Neutrophil, lymphocyte, monocyte, platelet, NLR, PLR, and MLR levels were divided into two groups according to optimum cut-off values determined by means of receiver operating characteristic (ROC) curve analysis and by maximizing the Youden index (sensitivity and specificity −1). Chi-square and Mann–Whitney *U* test were used in the comparison of categorical variables and of continuous variables, respectively, when comparing patients in whom pCR was or was not reached. Forward stepwise logistic regression (likelihood ratio) analysis was performed in order to detect independent factors predicting pCR. All statistical analyses were performed using IBM SPSS Statistics 20.0 software. A *P* value of <.05 was deemed statistically significant.

## 3 Results

A total of 130 patient records were reviewed retrospectively. The patients were divided into two groups: those in whom pCR was achieved after NAC and those in whom it was not achieved. pCR was achieved in 23.6% of the patients (n = 31). The clinicopathological features of those showing pCR and those not showing pCR were compared. Clinical tumor size T1 and T2 patients had higher pCR rates than T3 and T4 patients. Next, pCR rates were higher in ER-negative, PR-negative, and HER-2-positive patients; also, pCR was higher in patients with grade 2 and grade 3 tumors than in those with grade 1 tumors. In terms of molecular subgroups, pCR was higher in patients with luminal B-HER positivity, triple negativity, and HER-2 positivity (Table 1).

**Table 1:**
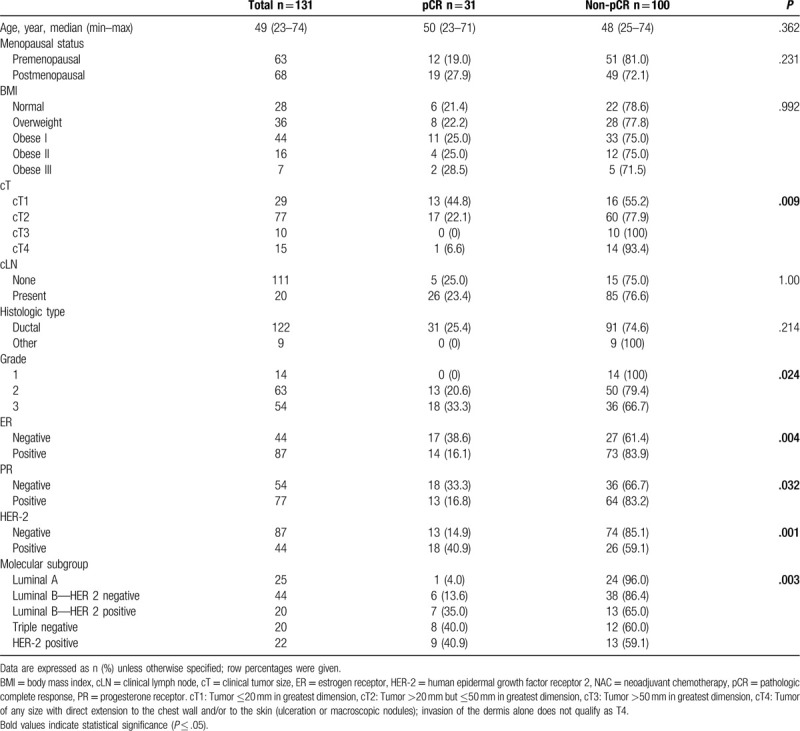
Clinicopathological features of the patients and their correlation with pCR after NAC.

**Figure 1:**
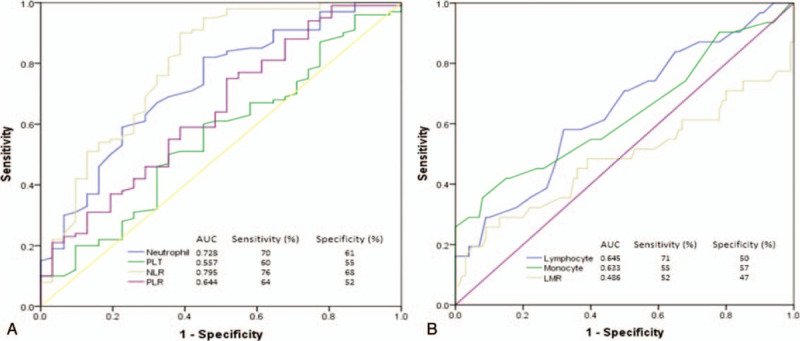
Receiver operative characteristic curves. (A) ROC curve for neutrophil, platelet (PLT), NLR, and PLR. (B) ROC curve for lymphocyte, monocyte, and LMR.

The optimum cut-off values for neutrophils, lymphocytes, platelets, NLR, PLR, and LMR at the time of diagnosis that would predict pCR were determined by ROC analysis. The cut-off values of 4150 μL, 2000 μL, 635 μL, 271 × 10^3^ μL, 1.95, 119, and 3.35 were reported, respectively, for neutrophils, lymphocytes, monocytes, platelets, NLR, PLR, and LMR. Sensitivity and specificity values were calculated, and the determined optimum cut-off values are shown in Figure 1A and Figure 1B.

The neutrophil, lymphocyte, and platelet counts and NLR, PLR, and LMR values of patients with and without pCR were compared (Table 2). The pCR rate was higher in patients with a low neutrophil count and in patients with either NLR or a high lymphocyte count (*P* values of, respectively, .002, <.001, and .040).

**Table 2:**
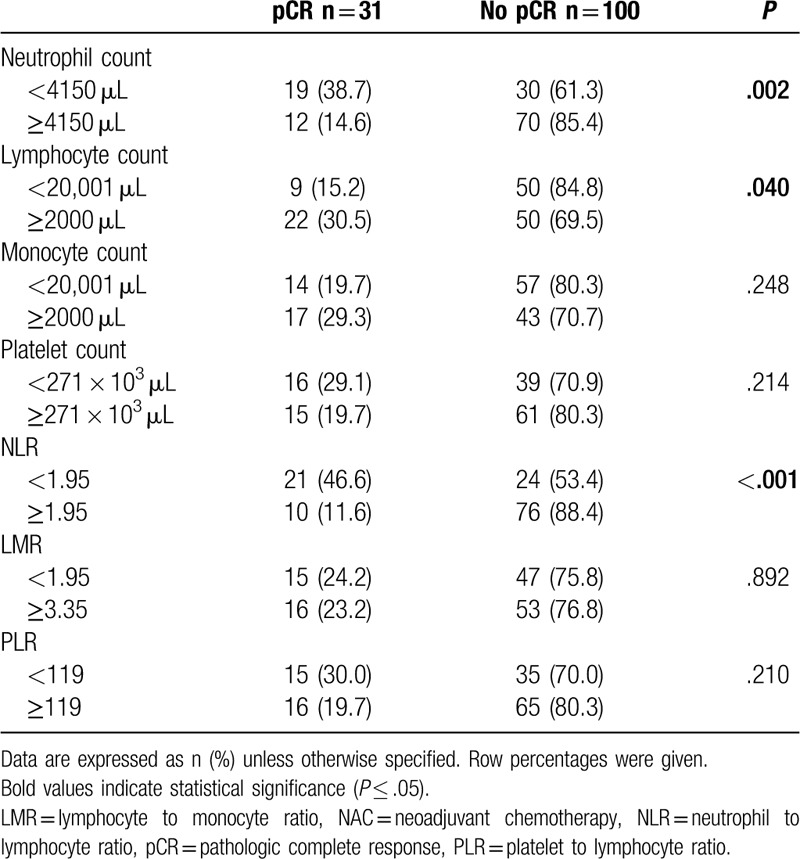
Hematologic findings and their correlation with pCR after NAC.

**Figure 2:**
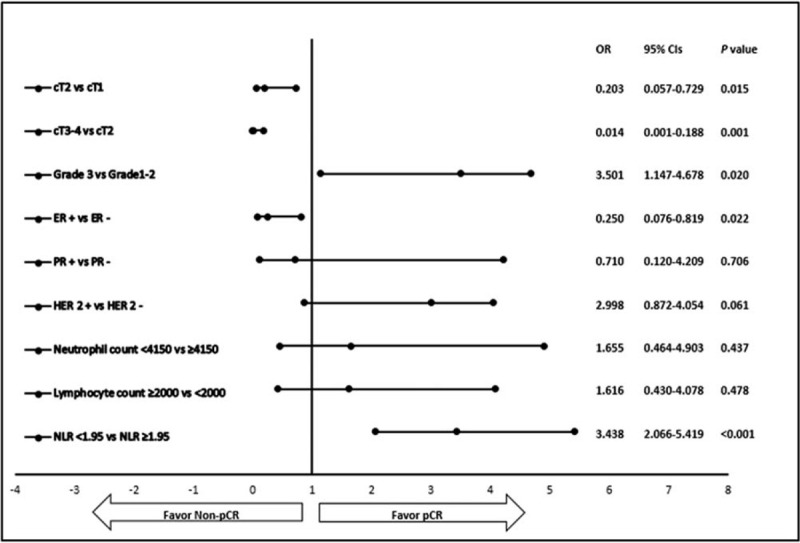
Forest plots.

The results of the multivariate regression analysis of clinical tumor size; tumor grade; ER, PR, and HER-2 status; neutrophil and lymphocyte counts; and NLR showed that cT1 and cT2, grade 3, ER negativity, and low NLR were independent predictive factors of pCR (Fig. 2). Better pCR was reported in patients with an NLR of <1.95 (OR: 3.438 95%CI: 2.066–5.419; *P* < .001).

## 4 Discussion

This study examined the factors that could predict pCR in patients who undergo NAC and surgery for LABC. Multivariate analysis revealed that NLR, cT, ER status, and tumor grade were independent predictive factors of pCR.

Studies have reported conflicting findings on the correlation between NLR and pCR in patients receiving NAC for LABC.^[16–19]^ Asano et al reported that among breast cancer patients receiving NAC, a higher rate of pCR was achieved in patients with a low NLR prior to therapy than in those with a high NLR.^[17]^ Rivas et al found in their study on luminal B breast cancer that NLR is significantly low in patients achieving pCR.^[16]^ Kim et al evaluated both NLR and PLR in their study and found that pCR was higher in patients with low NLR and/or PLR than in those with high values of the same (OR: 4.13, 95%CI: 1.49–11.43; *P* = .006).^[22]^ Suppan et al did not confirm the correlation between NLR and pCR.^[18]^ Eryilmaz et al stated that NLR does not predict pCR.^[19]^ Our study established that NLR is an independent predictive factor of pCR; while the pCR rate was 46.6% in patients with an NLR of <1.95, it was 11.6% in patients with an NLR of ≥1.95 (OR: 3.438, 95%CI: 2.066–5.419, *P* < .001).

One of the important factors affecting pCR after NAC in breast cancer is the hormone receptor status.^[4,23]^ Battisti et al determined a significantly high pCR rate in ER-negative patients receiving NAC as compared to in ER-positive patients.^[23]^ Minckwitz et al reported a pCR rate of 26% in ER-negative patients and of 7.6% in ER-positive patients (*P* < .001).^[4]^ Similar results were reported by Guarneri et al: 24% and 8% in ER-negative and ER-positive patients, respectively (*P* < .001).^[24]^ ER status was shown to be an independent predictive factor by Jarzab et al, and in their study, the pCR rate was 10.8% in ER-positive patients and 36.4% in ER-negative patients (OR: 0.41, 95%CI: 0.16–0.99; *P* = .047).^[25]^ Similarly, Villa et al found significantly high pCR rates in ER-negative patients (OR: 0.87, 95%CI: 0.82–0.93; *P* < .001).^[26]^ Our study showed, in line with the literature, that the pCR rate was low in ER-positive patients compared to in ER-negative patients and that ER status was an independent predictive of pCR (OR: 0.250, 95%CI: 0.076–0.819; *P* = .022).

Clinical tumor size at the time of diagnosis in LABC patients is known to be one of the factors predicting pCR after NAC, with a significant decrease in pCR as the cT increases.^[5]^ Choi et al found that cT is an independent predictive factor of pCR and the pCR rates are higher in cT1 patients.^[27]^ While cT was found to be associated with pCR in the univariate analysis conducted by Villa et al, multivariate analysis showed that cT was not an independent predictive of pCR.^[26]^

In our study, the pCR rate was significantly low in cT2 patients compared to in cT1 patients (OR: 0.203, 95%CI: 0.057–0.729; *P* = .015). Furthermore, the pCR rate was significantly low in cT3 and cT4 patients compared to in cT2 patients (OR: 0.014, 95%CI: 0.001–0.188; *P* = .001).

Tumor grade is one of the factors predicting pCR after NAC. A study by Minckwitz et al showed a higher pCR rate in patients with grade 3 tumors than in those with grade 1 or 2 tumors.^[4]^ Asano et al found high pCR rates in patients with high tumor grades, specifically in cases of triple-negative breast cancer.^[17]^ Jarzab et al confirmed that tumor grade is an independent predictive factor of pCR, and they reported pCR rates of 31.3% and 8.2% in patients with grade 3 tumors and grade 1 and 2 tumors, respectively (OR: 2.42, 95%CI: 1.16–5.33; *P* = .017).^[25]^ Villa et al found that higher pCR was obtained in patient with grade 3 tumor with positive axillary lymph node compared to in patients with grade 1 and 2 tumors (OR: 5.14, 95%CI: 1.09–24.1; *P* = .04).^[26]^ Our study indicated that tumor grade is an independent predictive factor of pCR, and the pCR rates were 33.3% and 20.3% in patients with grade 3 and grade 1 and 2 tumors, respectively (OR: 3.501, 95%CI: 1.147–4.678; *P* = .020).

There are some limitations to our study. The molecular subgroups could not be included in the multivariate analysis since the number of patients in our study was small. Instead, hormone receptor status, which is the basis for molecular subgroups, namely HER-2 status, and tumor grade were evaluated in the multivariate analysis. The small sample size did not allow us to separately examine the correlation between NLR and molecular subgroups.

## 5 Conclusion

Our study proposes that NLR is an independent predictive factor of pCR in LABC patients receiving NAC. We believe that the addition of NLR, which can be detected in a simple, quick, cheap, and practical manner, to the list of predictive clinicopathological features will increase the predictability of pCR in LABC patients assigned to NAC.

## Acknowledgments

The Authors thank our colleagues at the Diskapi Yildirim Beyazit Research and Education Hospital for their help and support.

## Author contributions

**Conceptualization:** Nuriye Ozdemir

**Data curation:** Tulay Eren, Cengiz Karacin, Gokhan Ucar, Yakup Ergun, Goksen Inanc Imamoglu

**Formal analysis:** Nuriye Ozdemir, Ozan Yazici

**Methodology:** Nuriye Ozdemir, Ozan Yazici

**Supervision:** Tulay Eren, Nuriye Ozdemir, Ozan Yazici

**Writing – original draft:** Tulay Eren, Cengiz Karacin, Nuriye Ozdemir, Ozan Yazici
